# Dataset on transcriptomic profiling of cholestatic liver injury in an *in vitro* and *in vivo* animal model

**DOI:** 10.1016/j.dib.2020.106156

**Published:** 2020-08-07

**Authors:** Eva Gijbels, Lindsey Devisscher, Mathieu Vinken

**Affiliations:** a Department of *In Vitro* Toxicology and Dermato-Cosmetology, Vrije Universiteit Brussel, Laarbeeklaan 103, 1090 Brussels, Belgium; b Basic and Applied Medical Sciences, Gut-Liver Immunopharmacology Unit, Faculty of Medicine and Health Sciences, Ghent University, Corneel Heymanslaan 10, 9000 Ghent, Belgium

**Keywords:** Bile duct ligation, Drug-induced cholestasis, Human hepatoma HepaRG cells, Microarray, Transcriptomics

## Abstract

The transcriptomic dataset (whole genome microarray Affymetrix Human U133 plus 2.0 and Affymetrix Mouse Genome 430 2.0) presented in this paper describes the differential gene expression profile of a human *in vitro* model of drug-induced cholestasis and a well-known mouse *in vivo* model of cholestasis. The *in vitro* model consists of human hepatoma HepaRG cells in monolayer configuration exposed to 3 different cholestatic drugs with or without bile acids. For *in vivo* modelling of cholestasis, mice were subjected to bile duct ligation surgery. Consecutive normalization, summarization and background adjustments have been made by means of Robust Multichip Average Express software.

**Specifications Table**

**Table:** 

*Subject*	Pharmacology, Toxicology and Pharmaceutical Science: Toxicology; Medicine and Dentistry: Hepatology
*Specific subject area*	Cholestatic liver injury
*Type of data*	Raw data
*How data were acquired*	Affymetrix GeneChip Human Genome U133 plus 2.0 array (ThermoFisher, Belgium) Affymetrix GeneChip Mouse Genome 430 2.0 array (ThermoFisher, Belgium) Ingenuity Pathway Analysis (IPA) (Qiagen, Belgium) Transcriptome Analysis Console (TAC) (ThermoFisher, Belgium)
*Data format*	Raw (.CEL), normalized and analyzed
*Parameters for data collection*	Male 8-weeks-old Sv129 mice (Harlan, The Netherlands) were housed in the animal facility of the Faculty of Medicine and Health Sciences (Ghent University, Belgium). Mice were allowed to acclimatize for at least 1 week*prior* to experiments. Care was given in accordance with the Federation for Laboratory Animal Science Associations guidelines and the national guidelines for animal protection. The animal protocols used in this study were evaluated and approved by the Ethical Committee of Experimental Animals at the Faculty of Medicine and Health Sciences, Ghent University, Belgium (ECD 15/36). Cholestasis was induced by performing bile duct ligation (BDL) surgery as previously described [1]. Control mice were sham operated, whereby the common bile duct was isolated, but not ligated. Liver samples were collected 6 weeks post-surgery. Cryopreserved differentiated HepaRG cells (Biopredic International, France) were cultured following manufacturer's instructions (Biopredic International, France). Hereafter, HepaRG cells were exposed to 60 µM atazanavir, 20 µM cyclosporin A and 30 µM nefazodone. A 50 times concentrated mixture of 5 bile acids (*i.e.* 66 µM glycochenodeoxycholic acid, 20 µM deoxycholic acid, 19.5 µM chenodeoxycholic acid, 19 µM glycodeoxycholic acid, and 17.5 µM glycocholic acid) was included in the cell culture medium of HepaRG cells from day 7 after seeding in combination with the drug. Incubations with drugs were routinely carried out for 72 h with daily renewal of cell culture media, including a 50 times concentrated bile acid mixture and drugs. Dimethyl sulfoxide (DMSO) treated HepaRG cells served as control. All conditions contained a final DMSO concentration of 0.25%. All compounds were purchased from Sigma Aldrich, Belgium.
*Description of data collection*	Total RNA was extracted from HepaRG cell culture samples that were treated with atazanavir, cyclosporin A and nefazodone in the absence or presence of a 50 times concentrated mixture of bile acids (*i.e.* ATA 60 µM, ATA + BA 60 µM, CsA 20 µM, CsA + BA 20 µM, NEFA 30 µM and NEFA + BA 30 µM), as well as from controls solely exposed to the 50 times concentrated bile acid mixture of bile acids and/or identical DMSO concentration (*i.e.* BA and CTL, respectively). For each condition samples were collected from 3 separate HepaRG batches (*n* = 3). Similarly, total RNA was extracted from liver samples of mice that underwent bile duct ligation (*i.e.* BDL) and sham surgery (*i.e.* CTL). Liver samples were collected from 6 BDL mice and 6 CTL mice (*n* = 6). Quantification and purity of the isolated RNA were determined *via* spectrophotometric analysis with a Nanodrop spectrophotometer (ThermoFisher Scientific, Belgium). Whole genome expression analysis was performed using microarray technologies (Affymetrix, Germany).
*Data source location*	Department of *In Vitro* Toxicology and Dermato-Cosmetology, Vrije Universiteit Brussel, Jette, Belgium.
*Data accessibility*	Raw data is available at the Gene Expression Omnibus (GEO) from The National Center for Biotechnology Information (NCBI) with access number GSE152494. https://www.ncbi.nlm.nih.gov/geo/query/acc.cgi?acc=GSE152494
*Related research article*	E. Gijbels, V. Vilas‐Boas, P. Annaert, T. Vanhaecke, L. Devisscher, M. Vinken, Robustness testing and optimization of an adverse outcome pathway on cholestatic liver injury. *Arch Toxicol* 94 (4): 1151–1172 (2020). 10.1007/s00204–020–02691–9[3]

## Value of the data

• The data provide the transcriptomic signature of drug-induced cholestasis *in vitro* and obstructive cholestasis *in vivo*.
• The data can support further research of the mechanistic basis of different types of cholestasis.
• Comparison of the transcriptomic signatures of other types of cholestasis will shed new light onto similar and dissimilar features of cholestasis types, which may serve as foundation for novel diagnostic strategies.
• The data assist in the assessment of the robustness of an adverse outcome pathway on cholestatic liver injury.

## Data description

Raw data are provided from whole genome transcriptomic analysis performed *via* microarray on 3 different batches of HepaRG cells exposed to 3 different cholestatic drugs, atazanavir (*i.e.* datasets ATA 60 µM 1-3), cyclosporin A (*i.e.* datasets CsA 20 µM 1-3) and nefazodone (*i.e.* datasets NEFA 30 µM 1-3) in absence or presence of a 50 times concentrated bile acid mixture (*i.e.* datasets ATA 60 µM + BA 1-3; CsA 20 µM + BA 1-3 and NEFA 30 µM + BA 1-3). Controls consist of HepaRG cells solely exposed to the vehicle (*i.e.* datasets CTL 1-3) and the 50 times concentrated bile acid mixture (*i.e.* datasets BA 1-3).

In parallel, whole genome transcriptomic data was also achieved from bile duct ligated mice (*i.e.* datasets CBDL1-6) and sham mice (*i.e.* datasets SHAM1-6). Data was obtained using Affymetrix Human Genome U133 plus 2.0 and Affymetrix Mouse Genome 430 2.0, which, in turn, were processed by the software program Robust Multichip Average (RMA) Express.

## Experimental design, materials and methods

Male 8-weeks-old Sv129 mice were subjected to bile duct ligation surgery. In brief, animals were anaesthetized with isoflurane inhalation (Isoflo, Abbott, Belgium). Once fully sedated, a midline abdominal incision was made, after which the common bile duct could be isolated and ligated between 2 knots of non-resorbable suture (Silkan 5/0, Braun Aesculap, Germany), as previously described [1]. Control mice were sham operated, whereby the common bile duct was isolated, but not ligated. Liver samples were collected 6 weeks post-surgery.

Cryopreserved differentiated HepaRG cells (Biopredic International, France) were thawed and seeded in basal hepatic medium supplemented with thaw seed and general purpose medium (Biopredic International, France) onto rat tail collagen (0.1mg/ml) (Corning, Sigma Aldrich, Belgium) coated 24-well plates following manufacturer's instructions. Hereafter, HepaRG cells needed to be refreshed every 2–3 days with basal hepatic medium supplemented with maintenance and metabolism medium (Biopredic International, France). At day 7, HepaRG cells were exposed to cholestatic concentrations of atazanavir (60 µM), cyclosporin A (20 µM), and nefazodone (30 µM) (Sigma Aldrich, Belgium) with or without a 50 times concentrated bile acid mixture (*i.e.* 66 µM glycochenodeoxycholic acid, 20 µM deoxycholic acid, 19.5 µM chenodeoxycholic acid, 19 µM glycodeoxycholic acid, and 17.5 µM glycocholic acid (Sigma Aldrich, Belgium)) added to the cell culture medium. Stock solutions were made of the drugs and bile acids in DMSO. The final incubation solutions were prepared *ex tempore* by diluting the stock solutions with basal hepatic medium supplemented with induction serum-free medium (Biopredic International, France) and contained a final DMSO concentration of 0.25%. After 72 h of exposure, samples were collected for RNA isolation by aspirating the cell culture medium and adding lysis buffer (lysis solution with 1% β-mercaptol) directly to the well (Qiagen, Belgium). Total RNA extraction (Qiagen, Belgium) was done according to manufacturer's instructions to both *in vitro* and *in vivo* samples. Quantification and purity of the isolated RNA were determined by means of spectrophotometric analysis with a NanoDrop® ND-100 Spectrophotometer (ThermoFisher Scientific, Belgium). A cut-off ratio between 1.8 and 2.1 for the absorption at 260/280 nm was used for assessing purity.

Whole genome expression analysis was performed using microarray technologies from Affymetrix (Germany) as previously described [2]. For this purpose, 100 ng total RNA per sample was amplified using a GeneChip 3′IVT Express Kit following manufacturer's instructions (Affymetrix, Germany). Amplified RNA was purified with magnetic beads and 15 mg biotin-amplified RNA was treated with fragmentation reagent. Then, 12.5 μg of fragmented amplified RNA was hybridized to Affymetrix Human genome U133 plus 2.0 GeneChip and Affymetrix Mouse Genome 430 2.0 GeneChip. Subsequently, the chips were placed in a GeneChip Hybridization Oven 645 (Affymetrix, Germany) following manufacturer's instructions. After incubation, the arrays were washed with GeneChip Fluidics Station 450 (Affymetrix, Germany) and stained with Affymetrix HWS kit. Thereafter, stained arrays were scanned *via* an Affymetrix GeneChip Scanner 3000 7G. Hybridzation controls were performed using Affymetrix GCOS software. Normalization quality controls, such as scaling factors, background intensities, noise and raw Q-values, average intensities and present calls were done with RMA Express software and were all within the acceptable limits.

## Ethics statement

Performed animal experiments were approved by the Ethical Committee of Experimental Animals at the Faculty of Medicine and Health Sciences, Ghent University, Belgium (ECD 15/36).

## Declaration of Competing Interest

The authors declare that they have no known competing financial interests or personal relationships which have, or could be perceived to have, influenced the work reported in this article.

## Appendix Supplementary materials

Image, application 1
